# Effects of dietary garlic (*Allium sativum*) and papaya (*Carica papaya*) leaf powder on production performance, ruminal methanogen levels, gut parameters, and meat quality in goats

**DOI:** 10.14202/vetworld.2024.2659-2666

**Published:** 2024-11-28

**Authors:** Qurrat-Ul-Ain, Imtiaz Rabbani, Muhammad Afzal Rashid, Muhammad Shahbaz Yousaf, Wasim Shehzad, Habib Rehman

**Affiliations:** 1 Department of Physiology, University of Veterinary and Animal Sciences, 54000, Lahore, Pakistan; 2 Department of Animal Nutrition, University of Veterinary and Animal Sciences, 54000, Lahore, Pakistan; 3 Institute of Biochemistry and Biotechnology, University of Veterinary and Animal Sciences, 54000, Lahore, Pakistan

**Keywords:** Beetal goat, growth performance, methane emissions, short-circuit current

## Abstract

**Background and Aim:**

Several approaches have been employed to mitigate methane emissions from livestock, with varied results. This study evaluated the effects of shade-dried ground garlic leaf (GL) powder and papaya leaf (PL) powder as crop waste on feed intake, growth performance, ruminal microbial counts, gut epithelial barrier functions, and meat quality in goats.

**Materials and Methods:**

Forty male adult Beetal goats were randomly divided into five treatment groups: (1) Control (basal diet only); (2) basal diet supplemented with 6% bromodichloromethane (BCM); (3) basal diet supplemented with 30% GL powder; (4) basal diet supplemented with 26% PL powder; and (5) basal diet supplemented with 30% GL powder and 26% PL powder (GP).

**Results:**

Average weight gain, feed conversion ratio, fecal score, and albumin improved in the GP. Aspartate transferase increased significantly in BCM, GL, and PL and was insignificant in the GP group compared with the C group. There was a 13% decrease in methanogen count in PL compared with C, but this difference was not significant between BCM and GP. Ruminal bacteria and protozoa were lowest in GL. Ruminal papilla height and surface area increased in the supplemented groups compared with C (p < 0.05). *In vitro* experiments using isolated ruminal epithelia revealed a 39% increase in short-circuit current in GP compared with C (p < 0.05). For meat parameters, the pH 24 h decreased significantly in GL compared to BCM.

**Conclusion:**

Dietary supplementations with GL and PL alone or in combination improved growth parameters and gut performance and reduced rumen methanogen levels without altering meat quality parameters. Proper diet formulation and further research on other ruminants may help reduce greenhouse gas emissions from livestock.

## Introduction

The global population is growing at 1.1% annually, and it is expected to reach 9 billion by 2050 [1], broadening the gap between food requirements and supply. Therefore, developing innovative and cost-effective livestock production strategies is imperative to overcome the escalating protein demand [2, 3]. Ruminants, with diverse microbial populations of bacteria, fungi, protozoa, and archaea in their rumen, can convert renewable resources, such as grassland and crop residues, into food for humans. Ruminal methanogens contribute to the rumen’s digestive physiology by maintaining low H_2_ pressure and producing methane (CH_4_) through a hydrogenotrophic pathway. However, CH_4_ cannot be utilized by ruminants and is released into the atmosphere, making ruminants a contributor to greenhouse gas (GHG) emissions, including carbon dioxide (CO_2_), CH_4_, and nitrous oxide (N_2_O) [4]. Despite its lower atmospheric concentration, CH_4_ is over 25 times more potent contributor to global warming than CO_2_; hence, mitigating CH_4_ emissions from livestock remains a pressing challenge that demands sustainable and cost-effective solutions [5].

In Pakistan, the sustainability of the livestock sector relies heavily on 80 million goats, which play a crucial role in human-consumable food. Goats are generally reared by poor people with limited resources and ethical awareness. Therefore, the use of subtherapeutic antibiotics as growth promoters is prevalent [6]. However, rising awareness of antimicrobial resistance and its associated public health risks has led to a shift away from antibiotic use in animal farming, driving the exploration of alternative strategies [7]. In developing countries, suboptimal feed quality and reduced livestock efficiency contribute significantly to GHG emissions. Limited access to advanced methods and feed formulations commonly employed in industrialized nations to mitigate CH_4_ production further aggravates this challenge [5]. Previous studies [8–11] have shown that various strategies can effectively reduce CH_4_ emissions from ruminants. Among these, dietary manipulation using various natural phytochemicals is promising because of their limited harmful effects on hosts and consumers [12, 13]. Garlic (*Allium sativum*), with its various active compounds (allicin, diallyl sulfide, diallyl trisulfide, allyl mercaptan), vitamins, and minerals, is a rich source of phytochemicals (saponin, tannin, flavonoids, and alkaloids) [14]. Numerous studies have shown that garlic leaves exhibit antimicrobial, antioxidant, and immunomodulatory effects in livestock and poultry [15]. The *in vivo* use of garlic oil not only reduced CH_4_ emissions but also improved performance and carcass quality in lambs [16]. Similarly, *Carica papaya* leaves (PLs) harbor flavonoids and terpenoids with antimicrobial activity, particularly against methanogens [17].

Despite our knowledge of the medicinal properties and beneficial effects of garlic leaves (GLs) and PLs, our understanding of their effects alone or in combination as a dietary supplement in ruminants is limited. Therefore, it was hypothesized that the combination of both herbs would have a synergistic effect on the ruminant digestive physiology and modulation of microbial ecology. Therefore, this study investigated the effects of GL and PL on feed intake, growth performance, rumen methanogen ecology, rumen epithelial performance, and meat quality in goats.

## Materials and Methods

### Ethical approval

This study was approved by the Ethical Review Committee of the University of Veterinary and Animal Sciences (UVAS), Lahore, Pakistan (Approval no. DR/265).

### Study period and location

The study was conducted from December 2023 to March 2024 at the Small Ruminant Training Center, University of Veterinary and Animal Sciences, Lahore, Pakistan. The entire experimental period was 77 days, with the first 10 days as an adaptation period to a garlic and PL diet. The following 60 days were considered the treatment period for data collection, while the remaining 7 days were allocated to *in vitro* experiments with rumen epithelia and meat analyses.

### Animals, experimental design, diet, and management

Forty male Beetal goats aged 12 ± 1 month, weighing 28 ± 2.36 kg (mean ± standard deviation) were available at the research center, were vaccinated for *Clostridium perfringens* (Enterotoxaemia, VRI, Lahore, Pakistan) and contagious caprine pleuropneumonia (Lyophilized CPPV, VRI, Lahore, Pakistan), and were treated for internal parasitosis, ticks and mites by oral administration of doramectin (1 mL/50 kg body weight, Dectomax; Pfizer, Brooklyn, NY, USA). The animals were randomly assigned to the following five treatments (N = 8 goats/treatment): (1) Control, basal diet only (C), (2) basal diet supplemented with 6% bromochloromethane (BCM), (3) basal diet supplemented with 30% garlic leaf (GL) powder, (4) basal diet supplemented with 26% papaya leaf (PL) powder, and (5) basal diet supplemented with 30% GL powder and 26% PL powder (GP). For the entire research period, animals were housed in individual iron pens of 1.5 m × 1.4 m (length × width) bedded with sand and fed twice daily at 06:00 and 18:00 h. Feed remnants were measured to calculate feed conversion ratio (FCR), and clean fresh water was available *ad libitum*.

### Leaf collection and chemical analysis

Garlic (*A. sativum*) leaves were collected from commercial garlic farms in Wah Cantt (33.7715° N, 72.7511° E) and Kalar Kahar (32.7769° N, 72.7068° E), Pakistan. Healthy green garlic leaves were cleaned, cut into 2–3 cm segments, and shade dried. The dried leaves were ground and fed to the experimental animals by mixing them with a concentrated fraction of the feed [17]. Fresh leaves of *C*. *papaya* were procured from a papaya farm in Sialkot, Pakistan. The leaves were thoroughly washed and dried in shade. The dried leaves were ground to a fine powder using a grinder and preserved at 27 ° C in tightly sealed zipper plastic bags for feeding experimental animals [15]. Powdered garlic and papaya leaf samples were analyzed according to the Association of Official Agricultural Chemists (AOAC) [18] for dry matter (DM), moisture, crude protein, crude fiber, fat, ash, and nitrogen free extracts (NFEs) contents, as shown in Table-1.

**Table-1:** Chemical composition of feed, garlic leaves (GL), and papaya leaves (PL) powder.

Chemical composition (%)	Garlic	Papaya	Concentrate	Hay
Dry matter	94.73	93.16	87.75	89.81
Moisture	5.27	6.84	-	-
Crude protein	15.40	17.60	20.89	7.32
Crude fiber	28.30	25.20	6.67	28.25
Fat	6.20	9.20	3.64	2.02
Ash	19.43	14.66	7.73	6.42
NFES	30.67	33.34	-	-

The concentrate was composed of ground corn, soybean meal, cotton seed barn, wheat corn, fish meal, calcium phosphate, limestone, trace mineral salts, and vitamin premix, NFE=Nitrogen free extracts

### Performance and hemato-biochemical parameters

FCR was calculated as the ratio of DMI to average daily gain for each goat. Average weight gain (kg) and body condition score (BCS) were recorded weekly; the fecal score was determined daily using a 1–5 scoring system, with 1 being watery and 5 being hard pallet. At the completion of the experiment, blood samples were collected from the jugular vein in a vacutainer, centrifuged at 2500× *g* for 20 min to obtain serum, and stored at –20°C until analysis. Glucose, total protein, albumin, globulin, cholesterol, blood urea nitrogen, creatinine, alanine transaminase, aspartate transferase (AST), and NEFA levels were measured using a calorimetric kit on an EPOC™ microplate spectrophotometer (Biotek Instruments Inc., Winooski, VT, USA) according to the manufacturer’s instructions.

### DNA extraction for reverse transcription polymerase chain reaction

Filtered rumen samples from experimental goats were harvested for DNA isolation. Extraction was performed on 20 mg samples using a high-throughput manual method. Cetyltrimethylammonium bromide (CTAB) lysis buffer (110 μL) was added to each rumen fluid sample, which was incubated at 65°C for 60 min. Each sample was extracted twice with 110 μL of phenol: chloroform:isoamyl alcohol (25:24:1, pH = 6.7; Sigma-Aldrich, USA) and once with 110 μL of chloroform: isoamyl alcohol (24:1; Sigma-Aldrich). After each extraction, samples were centrifuged at 3000× *g* for 60 min at 4°C, and the supernatant was transferred to wells containing 90 μL of isopropanol (Sigma-Aldrich) and 10 μL of 7.5 mM ammonium acetate. DNA was precipitated at –20°C overnight, followed by centrifugation of the samples at 3000× *g* for 60 min at 4°C. Next, three ethanol washes were performed by adding 110 μL of 70% (v/v) ethanol (Sigma-Aldrich) to each sample and centrifuging for 30 min at 3000× *g* at 4°C. Supernatants were discarded after each wash, and DNA pellets were air-dried before being resuspended in 50 μL of 75 mM TE buffer (pH = 8.0, Sigma–Aldrich). The yields and purities of the extracted DNA were assessed using a NanoDrop ND-2000 spectrophotometer (Thermo-scientific, Waltham, MA, USA) [19].

### Real-time polymerase chain reaction (PCR)

The extracted DNA samples were used as templates to quantify copy numbers of the 16S rRNA (for bacteria), methyl coenzyme-M reductase A (MCR A) gene (for methanogenic archaea), and 18S rRNA (for protozoa) using real-time quantitative PCR. The primer sets are listed in Table-2. Real-time PCR analysis was conducted using a Rotor-Gene Q (Qiagen, Hilden, Germany) real-time PCR cycler. Two μL of DNA extract were added to the amplification reaction (20 μL) containing 0.1 μL of 100 μM of each primer and 10 μL of Maxima SYBR Green qPCR 2X Master Mix (Thermo Fisher Scientific). The cycling conditions were 95°C for 10 min: 40 cycles of 95°C for 10 s, 60°C for 30 s, and 72°C for 30s. The threshold cycle of each sample was determined during the exponential amplification phase.

**Table-2:** Polymerase chain reaction (PCR) primers for real time PCR assay.

Microorganism	Sequence 5’--3’	Base pair
Total Bacteria	Forward: GTGSTGCAYGGYTGTCGTCA	130
Total Bacteria	Reverse: ACGTCRTCCMCACCTTCCTC	
Total Protozoa	Forward: GCTTTCGWTGGTAGTGTATT	223
Total Protozoa	Reverse: CTTGCCCTCYAATCGTWCT	
Total Methanogens	Forward: TTCGGTGGATCDCARAGRGC	140
Total Methanogens	Reverse: GBARGTCGWAWCCGTAGAATCC	

### Tissue sampling and handling

On day 60 of the experiment, randomly selected goats (N = 5 from each treatment group) were slaughtered at the Meat Science and Technology Department, UVAS. The visceral organs were removed, and the ventral segment of the rumen was isolated and stored in 10% formalin buffer for histological analysis. Another segment of the rumen with the muscularis layer stripped off was cut into pieces of 4 × 4 cm^2^ and transported in a buffer that contained (in mM) 70 NaCl, 0.4 NaH_2_PO_4_, 2.4 Na_2_HPO_4_, 5 KCl, 5 glucose, 30 Na-gluconate, 40 NMDG-Cl, 8 Hepes, 1.2 CaCl_2_, and 1.2 MgCl_2_, (pH 7.4 and osmolality 280 mOsm/L) to the Electrophysiology Lab., Department of Physiology, UVAS for electrophysiological measurements.

### Histomorphometry of the rumen

For histomorphometry, rumen samples were fixed in 10% neutral buffer formalin, dehydrated using graded alcohol, and cleared in xylol. After tissue sectioning, samples were stained with hematoxylin-eosin, and images were taken at 10× using an optical microscope (LX400, LABOMED, and the Netherlands) fitted with a digital camera (DC, 1355-F050, CMEX Euromex, and the Netherlands). Papilla length, width, and surface area were measured to assess the rumen parameters.

### Ussing chamber experiments with isolated ruminal epithelium

Electrical measurements were obtained using a computer-controlled voltage clamp device (Mussler Scientific Instruments, Aachen, Germany). Fluid resistance was measured and corrected before mounting the epithelium to determine the transepithelial potential difference (Pd). The isolated ruminal epithelia were then mounted between the two halves of the Ussing chamber with an exposed surface area of 3.14 cm^2^, and silicon rubber rings minimized the edge damage to the tissue. Buffer solution (16 mL) was added to both sides of the chamber and aerated with carbogen (95% oxygen and 5% CO_2_) while the temperature was maintained at 38°C. Tissues were allowed to equilibrate under an open circuit for 20 min and then short-circuited by clamping the voltage at 0 mV [20]. The changes in Pd, tissue conductance (Gt), and short circuit current (Isc) were calculated every 10 s. Isolated epithelia with Gt > 40 mS and Pd < 1 mV showed inconsistent responses to *in vitro* treatments and were therefore not included in this study.

### Meat analysis

Meat pH was recorded from the longissimus dorsi muscle approximately 25 mm from the medial line at a point over the 13^th^ rib. A penetrating probe (AOAC, 2005 method 981.12) of a portable pH meter (WTW, pH 3210 SET 2, Germany) recalibrated with buffer solutions of pH 4.0 and 7.0 was used to measure the pH at 24 h post-mortem. Meat color (L*, a*, and b*) was also measured using a chroma meter (Konica Minolta^®^ CR-410, Osaka, Japan) calibrated with a standard white tile (L* 95.18, a* 0.07, and b* 2.59), as instructed by the manufacturer. The meat steaks (2.0 cm thickness) were labeled and kept at 0°C–4°C for 12 days in vacuum-packed plastic bags (150 × 200, PA/PE90) using a vacuum packing machine (Multivac^®^ Baseline p100, Geprüfte Scherhert, AWG, Germany) and weighed using a digital compact weighing balance (SF-400, 7000 g) to measure drip loss. Weight differences were considered as drip losses [21].

Drip loss (%) = Initial weight Final weight/Initial weight × 100

The steaks were cooked until the core temperature was 72°C [22]. The core temperature was measured by inserting a digital thermometer probe (TP 101, −50°C to 300°C) into the steak. Warner-Bratzler shear force (WBSF) was determined using a texture analyzer (TA; XT plus^®^ Stable Microsystems texture analyzer, Godalming, UK). The cooked meat samples were cooled to 0°C–4°C for 40 min before testing. They were cut into 6 cm long, 1 cm tall, and 1 cm wide muscle strips. WBSF values were recorded in Newtons per centimeter square (N/cm^2^).

### Statistical analysis

Statistical analyses were performed using SPSS Version 20 (IBM SPSS, Chicago, IL, USA). The normal distribution of data was checked using the Kolmogorov–Smirnov test. Data are presented as mean ± standard error of the mean and analyzed using a one-way analysis of variance followed by Tukey’s tests. The level of significance was considered at p < 0.05. Graphical representation of data was conducted using a Sigma plot Version 12 (Grafiti LLC, London, UK).

## Results

### Animal performance parameters

The average weight gain (AWG) showed improvement in all treatment groups; however, it was significantly higher in GL and GP groups than in C group. In addition, FCR was improved in all treatment groups (p < 0.05) compared to the C group. In contrast, BCS was not significant between the C group and all treatment groups. For serum biochemistry analysis, albumin was significantly increased in GP compared to C, BCM, and GL. AST levels were higher in BCM, GL, and PL (p < 0.05), but the difference between C and GP was not significant. None of the other parameters significantly differed among the treatment groups (Table-3).

**Table-3:** Effect of dietary supplementation of garlic leaves (GL), papaya leaves (PL), and combination (GP) on anthropometric parameters.

Parameters	Group				

	C	BCM	GL	PL	GP
Performance					
AWG (kg)	0.65 ± 0.08^b^	1.35 ± 0.39^ab^	1.85 ± 0.28^a^	1.52 ± 0.18^ab^	2.150 ± 0.59^a^
FCR (DMI/WG*)	0.87 ± 0.11^a^	0.49 ± 0.11^b^	0.32 ± 0.0 ^b^	0.37 ± 0.04^b^	0.36 ± 0.14^b^
BCS	1.00 ± 0.01	1.11 ± 0.42	1.02 ± 0.18	1.02 ± 0.17	1.12 ± 0.43
Fecal score	2.01 ± 0.01^b^	3.12 ± 0.48^ab^	3.50 ± 0.29^ab^	3.75 ± 0.25^ab^	4.03 ± 0.70^a^
Serum biochemistry					
Glucose (mg/dL)	61.30 ± 3.16	64.60 ± 4.89	67.90 ± 3.13	64.90 ± 2.71	69.01 ± 1.51
Total protein (g/dL)	9.20 ± 0.81	8.90 ± 0.60	8.70 ± 0.55	8.70 ± 0.36	9.20 ± 0.34
Albumin (g/dL)	2.59 ± 0.15^b^	2.98 ± 0.31^b^	2.96 ± 0.26^b^	3.24 ± 0.23^ab^	3.78 ± 0.10^a^
Globulin (g/dL)	6.60 ± 0.79	5.91 ± 0.66	5.73 ± 0.59	5.45 ± 0.22	5.41 ± 0.34
Cholesterol (mg/dL)	83.20 ± 1.77	75.50 ± 3.25	79.50 ± 2.15	76.50 ± 2.58	76.30 ± 2.07
BUN (mg/dL)	25.60 ± 0.51	24.10 ± 0.76	23.20 ± 0.46	25.60 ± 0.52	25.10 ± 0.87
Creatinine (mg/dL)	0.73 ± 0.01	0.70 ± 0.01	0.74 ± 0.01	0.73 ± 0.01	0.72 ± 0.00
ALT (U/L)	10.90 ± 0.41	10.40 ± 1.10	11.40 ± 0.64	11.10 ± 0.47	10.60 ± 0.62
AST (U/L)	23.30 ± 1.54^b^	31.20 ± 0.64^a^	30.70 ± 1.90^a^	31.01 ± 0.62^a^	25.30 ± 1.54^ab^
NEFA (mmol/L) 1	0.47 ± 0.08	0.64 ± 0.02	0.66 ± 0.01	0.61 ± 0.06	0.66 ± 0.02

Means with different superscripts in the same row indicate a significant difference (p < 0.05); BCM=Bromochloromethane, AWG=Average weight gain, FCR=Feed conversion ratio (dry matter intake (Kg)/weight gain (Kg),

* =Data not shown, BCS=Body condition score, ALT=Alanine transaminase, AST=Aspartate transaminase, NEFA=Non-esterified fatty acids

### Rumen microbial count

Real-time PCR was conducted to assess alterations in the microbial count. There were significant decreases in bacterial, protozoal, and methanogen counts in GL and PL compared to C and BCM. The bacterial count was higher in BCM (p < 0.05) than in C; however, no significant differences were observed between the C and GP groups. Dietary treatment with GP increased the protozoal count, whereas GL and PL alone reduced it (p < 0.05). For the methanogens, dietary treatment resulted in an overall decrease. There was a 13% decrease in ruminal methanogens in (p < 0.05) PL group compared to the C group. At the same time, there was also a significant reduction in methanogen levels in the BCM group compared to the C group. However, the methanogen count was not significantly different between GP and BCM (Table-4).

**Table-4:** Microbial number in the rumen contents of goats supplemented with garlic leaves (GL), papaya leaves (PL), and combination (GP).

Log_10_ cells/µL	Groups				

	C	BCM	GL	PL	GP
Bacteria	1.55 ± 0.21^b^	1.59 ± 0.11^a^	1.21 ± 0.16^d^	1.44 ± 0.21^c^	1.56 ± 0.17^b^
Protozoa	1.32 ± 0.17^c^	1.41 ± 0.12^b^	0.96 ± 0.23^e^	1.02 ± 0.23^d^	1.54 ± 0.16^a^
Methanognes	1.83 ± 0.12^a^	1.70 ± 0.19^b^	1.65 ± 0.27^c^	1.58 ± 0.18^d^	1.72 ± 0.28^b^

Means with different superscripts in the same row indicate a significant difference (p < 0.05). BCM=Bromochloromethane

### Ruminal histomorphometry

The effects of garlic and papaya leaf dietary supplementation on the histomorphometric analysis of rumen epithelium are summarized in Table-5. Dietary supplementation with GL, PL, or GP significantly increased papillae height (p < 0.05) compared to C. In particular, the papillae height increased by approximately 52% in GL compared to C (p < 0.05). However, BCM or dietary supplementation with GL, PL, or GP had no significant effect on papillae width (p > 0.05). The ruminal epithelial surface area was significantly increased in the GL and GP groups compared to the C and BCM groups.

**Table-5:** Histomorphometry of goat rumen (μm) supplemented with garlic leaves (GL), papaya leaves (PL), and combination (GP).

Parameters	Group				

	C	BCM	GL	PL	GP
Papillae height	1226.75 ± 50.78^d^	1493.31 ± 77.22^c,d^	1865.45 ± 81.61^a^	1582.32 ± 78.27^a,c^	1679.82 ± 83.78^a,b,c^
Papillae width	351.93 ± 10.44	396.80 ± 16.95	373.86 ± 11.94	351.15 ± 9.18	393.11 ± 13.93
Surface area (mm^2^)	1.43 ± 0.84^c,b^	1.91 ± 0.12^b,c^	2.26 ± 0.18^a^	1.81 ± 0.15^a,b,c^	2.21 ± 0.34^a^

Means with different superscripts in the same row indicate a significant difference (p < 0.05). BCM=Bromochloromethane

### Electrophysiological measurements

**Figure-1:**
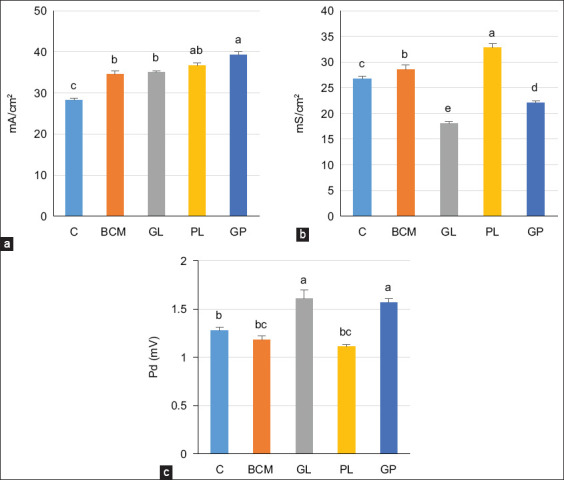
Effect of garlic and papaya leaf supplementation on electrophysiological parameters of isolated ruminal epithelia (n = 5 goats/treatment). (a) Short circuit current (Isc), (b) tissue conductance (Gt), and (c) transepithelial potential difference (Pd). Mean values in columns bearing different superscripts differ significantly (p < 0.05).

Measurements of electrophysiological parameters revealed differences between the experimental groups. GL and GP significantly increased Pd compared to C and BCM (p < 0.05). There was a 40% increase in Isc in GP compared to C, and it was significantly higher in BCM and GL. Gt significantly differed among all treatment groups, with the highest in PL and the lowest in GL (Figure-1).

### Meat analysis

The meat pH (24 h) was significantly lower in GL than in BCM (p < 0.05) but was not significantly different from the rest of the treatment groups. All other parameters, including drip loss, shear force, redness, lightness, and yellowness, remained unaffected by dietary supplementation (Table-6).

**Table-6:** Meat parameters of goat supplemented with garlic leaves (GL), papaya leaves (PL), and combination (GP).

Parameters	Groups				

	C	BCM	GL	PL	GP
pH 24h	6.14 ± 0.14^ab^	6.57 ± 0.0^a^	6.02 ± 0.1^b^	6.24 ± 0.2^ab^	6.18 ± 0.1^ab^
Drip loss	1.21 ± 0.01	1.13 ± 0.02	1.57 ± 0.07	1.13 ± 0.01	1.30 ± 0.03
Shear force	62.51 ± 0.72	63.23 ± 0.52	59.29 ± 3.75	55.51 ± 6.11	63.43 ± 0.32
Redness (a*)	18.66 ± 0.32	19.31 ± 0.61	18.15 ± 0.42	18.19 ± 0.32	18.62 ± 0.23
Lightness (L*)	42.19 ± 2.68	42.89 ± 1.97	44.05 ± 2.04	42.45 ± 3.19	42.51 ± 2.42
Yellowness (b*)	5.28 ± 0.21	6.01 ± 0.43	4.89 ± 0.58	3.82 ± 0.37	6.59 ± 0.27

^ab^Mean with different superscripts in the same row indicates a significant difference (p < 0.05); BCM=Bromochloromethane

## Discussion

### Performance parameters

Garlic and papaya leaves are often discarded after harvesting garlic bulbs or papaya fruits and are considered waste. However, garlic and papaya leaves contain high levels of bioactive compounds that may benefit animal health. CH_4_ emissions from livestock are a global concern. Therefore, this study aimed to evaluate the individual or combined effects of shade-dried garlic and PL on the performance, ruminal methanogen levels, epithelial parameters, and meat quality attributes of goats. Although a study by Pirmohammadi *et al*. [23] has shown the beneficial effects of garlic bulb supplementation, average daily gain, DM intake, and FCR are generally not affected. In our study, dietary supplementation with garlic and PL improved weight gain and FCR, a finding that is consistent with previous results for goats fed garlic flour [24].

However, our findings are in contrast to those of a previous study on the effects of garlic inclusion [25], where garlic inclusion >30 g/kg had a negative effect on fattening goats. Improvements in these parameters in our study could be due to the added advantage of GL over garlic bulb. A study by Panthee *et al*. [22] has shown that supplementation with garlic leaves improves nitrogen retention and microbial nitrogen supply in sheep without negatively affecting rumen fermentation characteristics. In contrast, garlic powder supplementation did not affect DM intake or FCR [26]. Contrary to our study, PL supplementation increased *Butyrivibrio* but did not affect the population of total ruminal bacteria, possibly due to the presence of phenols in PL, as reported by Jafari *et al*. [27].

### Biochemical parameters

Garlic and PL supplementation increased serum albumin levels by 45% compared to the control. Previous study by Kholif *et al*. [28] has also observed this trend, where lactating goats were supplemented with raw garlic. However, these findings differ from those of Pirmohammadi *et al*. [23], who observed reduced serum albumin and protein levels in pre-partum Mahabadi goats. A possible reason for this discrepancy might be the study design and the physiological status of the animals. In this study, garlic and papaya leaf supplementation increased AST levels by 34%, contrary to a previous study by Usur *et al*. [29] in which a decrease in AST was observed in kids supplemented with 3% garlic, possibly due to the beneficial effects of garlic as an antioxidant and hepatoprotective. However, since our study included GL supplementation, the anti-oxidant capacity appeared to be lower than that of the garlic bulb.

### Ruminal microbial counts

Garlic and papaya leaf supplementation significantly reduced the ruminal microbial count. Previous studies have shown that garlic supplementation can alter the composition of ruminal bacteria. While specific studies on garlic leaves are limited, garlic and its constituents have been reported to modify the rumen microbiome, leading to increased populations of beneficial bacteria and decreased populations of methanogens responsible for CH_4_ production [30, 31]. Our study demonstrated a 13% reduction in ruminal methanogens with papaya leaf supplementation, whereas GL supplementation reduced methanogens by 9%. The presence of organosulfur compounds in garlic, such as allicin, is believed to inhibit these methanogens, thus reducing CH_4_ output [22].

In contrast, papaya leaves contain bioactive compounds, such as tannins, that directly inhibit methanogenic archaea in the rumen. Previous studies [17, 27] have found that replacing one-fourth of a portion of alfalfa hay with papaya leaves significantly decreased methanogen concentration compared to the non-supplemented group. The tannin content of papaya leaves likely contributes to this direct inhibitory effect (2%–10%) on methanogens [17].

### Ruminal histomorphometry

Garlic and papaya leaf supplementation increased villus height and epithelial surface area. Although specific studies focusing on rumen histomorphometry are limited, garlic and papaya supplementation has been associated with positive effects on the histological structure of rumen [23]. Organosulfur compounds in garlic, such as allicin, and bioactive compounds in papaya leaves may enhance the health of the rumen epithelium, potentially leading to improved nutrient absorption and overall rumen function. By reducing methanogen populations and promoting beneficial bacteria, garlic and papaya leaf supplementation may help maintain a healthier rumen environment, which could be reflected in the histological structure [32, 33].

### Electrophysiological parameters

The electrophysiological parameters of isolated ruminal epithelia were improved in the supplemented groups. Our study revealed that PL supplementation increased Isc and Gt but decreased Pd. These findings are consistent with Ma *et al*. [34], who reported the same trend in goats supplemented with thiamine. In contrast, GL supplementation in our study decreased Gt and increased Pd, whereas Isc was improved in all supplemented groups compared to C (p < 0.05). An increase in Isc could be due to the change in volatile fatty acid concentration in the rumen of the supplemented groups and, consequently, increased activity of sodium-hydrogen exchanger 3 activity, which regulates intracellular pH [35].

### Meat parameters

Apart from pH 24 h in GL, the remaining meat parameters were unaffected in this study. These findings agree with Chen *et al*. [36] that garlic supplementation does not affect meat quality in ruminants. Dietary supplementation with papaya leaves in ruminants is limited. Most previous studies have suggested that papaya supplementation does not affect the carcass characteristics of broilers [37, 38]. However, the beneficial effects of papaya supplementation have also been reported [39].

## Conclusion

It can be concluded that while garlic bulbs and papaya fruits are acknowledged for their beneficial effects on animals, GL and PL should not be considered waste because their supplementation improves average weight gain, FCR, and ruminal parameters without affecting meat quality. In addition, garlic and papaya leaf supplementation can be a practical and potentially cost-effective approach to reducing CH_4_ emissions from livestock, as it reduces ruminal methanogens.

## Authors’ Contributions

QA and IR: Conceptualized and designed the study. MAR: Field trial with supplementation. IR: Supervised the study. WS: Execution and interpretation of the microbial assay. IR, MSY, and HR: Interpreted and critically revised the manuscript for intellectual input. MSY: Biochemical analysis. HR: Statistical analysis. IR: Reviewed and edited the manuscript. All authors have read and approved the final manuscript.
